# CK2.1, a bone morphogenetic protein receptor type Ia mimetic peptide, repairs cartilage in mice with destabilized medial meniscus

**DOI:** 10.1186/s13287-017-0537-y

**Published:** 2017-04-18

**Authors:** Hemanth Akkiraju, Padma Pradeepa Srinivasan, Xian Xu, Xinqiao Jia, Catherine B. Kirn Safran, Anja Nohe

**Affiliations:** 1 Department of Biological Sciences, University of Delaware, Newark, DE 19716 USA; 2 Helen F Graham Cancer center, Christiana Care, University of Delaware, Newark, DE 19716 USA; 3 Department of Material Sciences and Engineering, University of Delaware, Newark, DE 19716 USA; 4 Present address: NAL Pharmaceuticals Ltd, Monmouth Junction, NJ 08852 USA; 5 Quantitative Proteomics and Metabolomics Center, Columbia University, New York, NY 10027 USA

**Keywords:** Hyaluronic acid, Casein kinase II, Bone morphogenetic protein, Osteoarthritis, Cartilage

## Abstract

**Background:**

Osteoarthritis (OA) of the knee involves degeneration of articular cartilage of the diarthrodial joints. Current treatment options temporarily relieve the joint pain but do not restore the lost cartilage. We recently designed a novel bone morphogenetic protein receptor type I (BMPRI) mimetic peptide, CK2.1, that activates BMPRIa signaling in the absence of bone morphogenetic protein (BMP). Our previous research demonstrated that CK2.1 induced chondrogenesis in vitro and in vivo; however, it is unknown if CK2.1 restores damaged articular cartilage in vivo. In this study, we demonstrate that CK2.1 induced articular cartilage (AC) repair in an OA mouse model.

**Methods:**

We designed hyaluronic acid (HA)-based hydrogel particles (HGPs) that slowly release CK2.1. HGP-CK2.1 particles were tested for chondrogenic potency on pluripotent mesenchymal stem cells (C3H10T1/2 cells) and locally injected into the intra-articular capsule in mice with cartilage defects. C57BL/6J mice were operated on to destabilize the medial meniscus and these mice were kept for 6 weeks after surgery to sustain OA-like damage. Mice were then injected via the intra-articular capsule with HGP-CK2.1; 4 weeks after injection the mice were sacrificed and their femurs were analyzed for cartilage defects.

**Results:**

Immunohistochemical analysis of the cartilage demonstrated complete repair of the AC compared to sham-operated mice. Immunofluorescence analysis revealed collagen type IX production along with collagen type II in the AC of mice injected with HGP-CK2.1. Mice injected with phosphate-buffered saline (PBS) and HGP alone had greater collagen type X and osteocalcin production, in sharp contrast to those injected with HGP-CK2.1, indicating increased chondrocyte hypertrophy.

**Conclusions:**

Our results demonstrate that the slow release HGP-CK2.1 drives cartilage repair without the induction of chondrocyte hypertrophy. The peptide CK2.1 could be a powerful tool in understanding the signaling pathways contributing to the repair process, and also may be used as a potential therapeutic for treating degenerative cartilage diseases such as OA.

## Background

Articular cartilage (AC) is a viscoelastic connective tissue that covers the articular ends of the femoral bone and is essential for the free movement of the joints. It consists of chondrocytes that are responsible for the production of extracellular matrix (ECM) within this tissue. This ECM network maintains the load bearing properties for mechanical compression across the joint [1]. Osteoarthritis (OA) is a cartilage metabolic disease affecting 21.7 million people each year and is the eleventh highest contributor of disability and costs over $28.5 billion dollars a year [2–4]. In OA, the AC undergoes progressive loss of ECM, enhanced chondrocyte hypertrophy, and chondrocyte apoptosis loss [5]. Chondrogenic ECM is composed of proteoglycans, such as aggrecans, and collagens, including type II, type IX, and type XI, that form a cross-linked network to regulate its biological properties [6]. More importantly, AC is an avascular tissue and possesses poor tissue regenerative capacity. In addition, OA is accompanied by remodeling and sclerosis of the subchondral bone and formation of osteophytes [7].

Among many growth factors that influence the progression of OA, bone morphogenetic proteins (BMPs), such as BMP2, greatly accelerate the overall loss of AC [8]. Interestingly, BMP2 is a potent growth factor with many pleiotropic functions including AC formation [9, 10]. BMP2 is also known to induce chondrocyte hypertrophy followed by cartilage calcification [10]. Therefore, BMP2 may not be valuable as a therapeutic for cartilage restoration in degenerative diseases such as OA.

BMP2 signals through binding to type I and type II serine/threonine kinase receptors. Upon ligand binding, the type I receptor is phosphorylated by the constitutively active type II receptor at the GS box (glycine/serine rich region) to initiate downstream signaling [11]. We previously reported that the protein casein kinase 2 (CK2) interacts with the bone morphogenetic protein receptor type Ia (BMPRIa) [11] and that loss of this interaction leads to the activation of BMP signaling in the absence of the ligand [11, 12]. Furthermore, we designed a peptide, CK2.1, that inhibits CK2 binding to BMPRIa to activate signaling downstream [11]. We previously demonstrated the potency of CK2.1-induced chondrogenesis in vitro and in vivo [13]. Here, we evaluate the potential of the peptide in cartilage repair in vivo. For minimal invasive delivery of the peptide to the damaged cartilage, we conjugated the peptide CK2.1 to hyaluronic acid (HA)-based hydrogel particles (HGPs) through hydrolytically degradable ester linkages (HGP-CK2.1). This allows the slow release of the CK2.1 over a 7-day period. Destabilization of the medial meniscus (DMM) surgery was performed to induce OA-like changes in mice AC and to test the cartilage regeneration potential of CK2.1. Six weeks after DMM surgery, HGP-CK2.1 was injected via the intra-articular route twice, once every 2 weeks, and mice were sacrificed 2 weeks after the last injection. Immunohistochemical analysis demonstrated a significant increase in collagen type II and type IX in mice treated with HGP-CK2.1, but not in the controls or mice injected with phosphate-buffered saline (PBS). While PBS-injected mice demonstrated a significant increase in collagen type X and osteocalcin production, HGP-CK2.1-injected mice did not. Our data show that localized intra-articular injections of HGP-CK2.1 restored the AC and stimulated cartilaginous ECM production.

## Methods

### Mice

All 10-week-old male C57BL/6 J mice were obtained from The Jackson Laboratory (Bar Harbor, ME, USA) and maintained under conventional housing conditions. The animal protocol was approved by the IACUC at the University of Delaware. Male mice of age 10 weeks (*n* = 6/group) were separated in four groups: HGP-CK2.1-, HGP-, and PBS-injected groups, and sham-operated mice.

### Surgical destabilization of the medial meniscus

Mice were anesthetized using isoflurane prior to and during surgery. All mice had their right knees shaved using an electric trimmer and wiped using alcohol swabs to sanitize the area of surgery. A 3-mm longitudinal incision extending from the distal patella to the tibial plateau was created. The joint capsule medial to the patellar tendon was incised with a #11 scalpel blade and the capsule was opened with micro-iris scissors. Dissection of the fat pad located over the intercondylar area provided visualization of the medial meniscus. An incision of the medial meniscotibial ligament was made at the tibial plateau to destabilize the meniscus [14]. Gauze dabbed with alcohol was used to stop any bleeding. The joint capsule was closed with a continuous 8-0 vicryl® suture and the skin was closed by the application of tissue adhesive. Mice were checked regularly for breathing movements, and antibiotics and pain killer were applied every 12 h during the first day following surgery, and monitored every day until the time of intra-articular injections. Mice were allowed to sustain OA-like damage to the cartilage 6 weeks post-surgery. HGP-CK2.1, HGP alone diluted in PBS, or PBS alone to a total volume of 6 μl was injected twice using a Hamilton syringe with a 30-gauge needle into the patellar cavity, once every 2 weeks, 6 weeks after DMM surgery.

### Design of peptides

Peptides were designed by our group as previously described [11]. A prosite search including patterns with high probability of occurrence on BMPRIa yielded possible CK2 phosphorylation sites located at amino acids 466–469 (SYED). The peptides were designed with the Antennapedia homeodomain signal sequence for cellular uptake and incorporated in one of these binding sites: CK2.1 (SYED). The peptides included several amino acid residues flanking each side [15].

### Preparation of CK2.1-conjugated HGPs

HA-based HGPs with a mean diameter of 10 μm were formulated by an inverse emulsion cross-linking technique, following previously reported procedures [16]. Separately, a heterobifunctional linker, containing a thiol-reactive acrylate group and an amine-reactive N-hydroxysuccinimide ester (NHS) with three lactic acid (LA) repeats, was synthesized according to reported methods [17]. Next, HA HGPs (10 mg) and the bifunctional linker (4.5 mg) were mixed in 2 ml DMSO and the reaction was allowed to proceed for 24 h at 40 °C under constant stirring. The modified particles were then washed thoroughly with water, ethanol, and acetone before being dried at 37 °C in the incubator overnight. Subsequently, the modified HGPs (10 mg) was added to 10 ml PBS containing 10 mg of the cysteine-tagged CK2.1 peptide (QIKIWFQNRRKWKKMVPSDPSYEDMGGC, GenScript). The reaction was allowed to proceed at room temperature for 24 h. The product, HGP-CK2.1, was washed thoroughly with PBS, isolated by centrifugation at 3000 rpm for 5 min, and finally reconstituted in PBS at the desired concentration. The peptide content in HGP-CK2.1 was determined by subtracting the unreacted peptide in the washing supernatant from that in feed, as quantified by UV-Vis spectrophotometer at 280 nm based on a standard curve of CK2.1 in PBS (62.5–1000 μg/ml). To prepare sterile particle formulations, HGPs were first suspended and sterilized in 70% (v/v) ethanol overnight before reacting with sterile-filtered (0.22 μm) CK2.1 peptide in PBS.

### In vitro release of CK2.1

HGP-CK2.1 (2 mg) was dispersed in PBS (0.5 ml) under constant rotation at 37 °C. At predetermined time points, the supernatant was collected by centrifugation (3000 rpm for 5 min), and the release medium was replenished with an equal amount of fresh PBS. The released peptide in PBS was quantified by UV-Vis spectrophotometer at 280 nm. The cumulative release was calculated as the total amount of CK2.1 peptide released into the medium at a particular time relative to the initial loading.

### Cell culture

C3H10T1/2 cells were purchased from American Type Culture Collection (CCL-26) (Manassas, VA, USA) and monolayer cultures were maintained in T-75 flasks grown in Dulbecco’s modified Eagle’s medium (DMEM; Mediatech, Manassas, VA, USA) supplemented with 10% (v/v) fetal bovine serum (FBS; Gemini Bioproducts, West Sacramento, CA, USA), 0.5% (v/v) l-glutamine (Mediatech), and 1% (v/v) penicillin/streptomycin (100 IU/ml penicillin, 100 μg/ml streptomycin; Fisher Scientific, Pittsburg, PA, USA). Cultures were incubated at 37 °C and 5% CO_2_ in air, and cells were passaged at 90% confluency with 0.05% (v/v) trypsin-EDTA (Gemini Bioproducts).

### Alcian blue staining

C3H10T1/2 cells were seeded at 1 × 10^7^ cells/ml and plated as a 10-μl micromass culture in a 1.9 cm^2^ 24-well plate (Nunc, Rocskilde, Denmark). Cells were supplemented with DMEM with 10% (v/v) FBS and incubated at 37 °C and 5% CO_2_. Cells were then stimulated with recombinant BMP2 (40 nM; GenScript, Piscataway, NJ, USA) or HGP-CK2.1 (5 nM or 10 nM or 30 nM or 50 nM/day release concentration).

Seven days after stimulation, cultures were fixed using 10% (v/v) neutral-buffered formalin (pH 7.4) mixed with 0.05% wt/v cetylpyridinium chloride for 20 min at room temperature. Cells were rinsed three times with 3% (v/v) glacial acetic acid (pH 1.0), and stained using 0.5% (w/v) Alcian blue 8-GX stain (Life line, Walkersville, MD, USA) overnight. After staining, cultures were rinsed with 3% (v/v) glacial acetic acid (pH 1.0) and air dried. Stained cultures were viewed under an inverted light microscope (Nikon, TMS-f) using 20× magnification and the collected images were analyzed and quantified with ImageJ software (NIH, Bethesda, USA) [18].

### Histological scoring

Two weeks after the last intra-articular injections, the mice were sacrificed and dissected femurs were fixed in 10% (v/v) neutral buffered formalin (Sigma Aldrich, St. Louis, MO, USA) and decalcified for 5 days in 5% (v/v) formic acid in 10% (w/v) sodium citrate (Sigma Aldrich). Femur samples (*n* = 6/group) were paraffin embedded and sectioned at 6-μm thickness. Sectioned slide samples were stained using Safranin O and fast green staining [19, 20]. Scoring was performed in four compartments of the knee using a modified semiquantitative scoring scale as described previously [14]. The scoring analysis used in this study was: score 0 = normal cartilage, score 0.5 = loss of safranin O staining with a normal articular surface, score 1 = small fibrillations or roughened articular surface, and score 2 = fibrillations extending into the superficial lamina. For each knee analyzed, 12 slides representing the entire joint were blinded and scored by two independent observers.

### Immunostaining

Sectioned femur samples (*n* = 6/group) (pre-treated in xylene for 10 min to clear away the paraffin) were incubated with testicular hyaluronidase for 30 min to expose collagen epitopes. The samples were immunofluorescently labeled for 1 h at room temperature either with rabbit polyclonal IgG collagen type II (10 μg/ml; ab34712; Abcam, UK) followed by Alexa 488 donkey anti rabbit IgG (2 μg/ml; Invitrogen, Eugene, OR, USA) or rabbit polyclonal collagen IX (10 μg/ml; Abcam) followed by Alexa 647 goat anti-rabbit IgG (2 μg/ml; Invitrogen) or Rabbit (Rb) pAb collagen X (10 μg/ml; ab58632; Abcam) followed by Alexa fluor 488 donkey anti-rabbit (2 μg/ml; Invitrogen) or rabbit polyclonal IgG osteocalcin (10 μg/ml; Santa Cruz Biotechnology, CA, USA) followed by Alexa fluor 488 donkey anti-rabbit (2 μg/ml; Invitrogen). Antibodies were diluted in 3% (w/v) bovine serum albumin (BSA). The nuclear stain bisbenzimide (Sigma Aldrich; Hoechst dye No. 33258, dissolved in H_2_O) was administered for 5 min and coverslips were mounted on slides using Airvol as described previously [21, 22]. Images were taken (*n* = 8 image sections/sample) on the Zeiss 780 confocal with a 20× objective (0.75NA, Beam Splitter (MBS) 458/514/561/633, 5% laser output, and (MBS) 405, 2% laser output). Images were quantified using ImageJ (NIH, Bethesda).

### Statistical data analysis

All data presented were analyzed using single factor analysis of variance (ANOVA), followed by Tukey-Kramer post-hoc test. All experiments were repeated three or more times and normalized to control. Error bars represent standard error of the mean (SEM), where * denotes statistical significance at *p* < 0.05 and ** denotes statistical significance at *p* < 0.01.

## Results

### HGPs release CK2.1 in a controlled manner

**Fig. 1:**
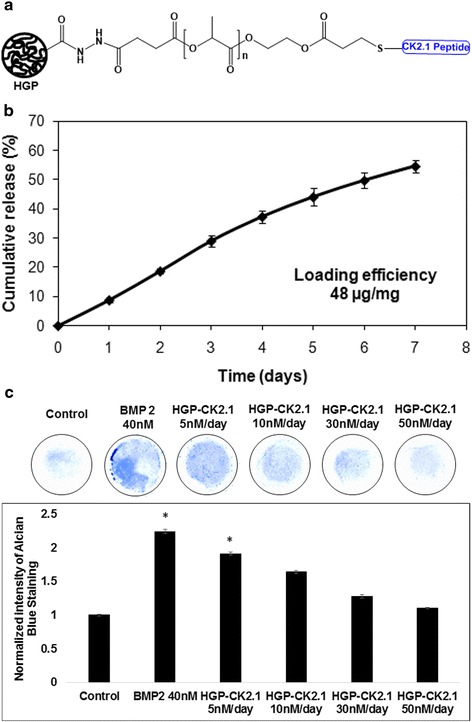
Synthesis of HGP-CK2.1 drug depots for sustained release. **a** Schematic illustration of CK2.1 peptide immobilized to HA HGPs via a hydrolytically degradable linker. **b** Cumulative release of CK2.1 from HA HGPs; peptide release was monitored by UV-Vis at 280 nm. Sustained release was achieved with +9.4 wt% release over days 0–4, and +5.3 wt% release over days 5–7. **c** C3H10T1/2 micromass cultures were treated with either BMP2 (40 nM) or HGP-CK2.1 (5 nM/day, or 10 nM/day, or 30 nM/day, or 50 nM/day release concentration) and stained with Alcian blue after 7 days. BMP2- and HGP-CK2.1-treated cells showed a significant increase in ECM containing proteoglycans. The mean of the samples was normalized to controls. Error bars represent SEM; *p < 0.05. *BMP* bone morphogenetic protein, *HGP* hydrogel particle

CK2.1 was covalently conjugated to the HGPs via Michael addition using cysteine-tagged peptide and acrylated HGPs (Fig. 1a). Approximately 48 μg of CK2.1 was conjugated to 1 mg of HGPs. The peptide was released (Fig. 1b) from the HGPs at a rate of +9.4 wt%/day from day 0 to day 4 and +5.3 wt%/day from day 4 to day 7. By day 7, when the experiment was terminated, a total of 54.6 wt% of the initially loaded peptide was released from the particles.

Next, we used C3H10T1/2 mesenchymal progenitor cells in micromass cultures to test the chondrogenic potency of the slow-release HGP-CK2.1. Our previous study demonstrated positive chondrogenic activity among C3H10T1/2 cells in micromass cultures stimulated with CK2.1 at concentrations ranging from CK2.1 at 100 nM (lowest) to 500 nM (highest) [13]. Therefore, C3H10T1/2 cells micromass cultures were stimulated with HGP-CK2.1 concentrations calculated based on CK2.1 total concentration of 50 nM or 100 nM or 300 nM or 500 nM. However, the release of CK2.1 from HGP is noted based on the cumulative release of the total amount of CK2.1 released from the initial loading. The adjusted HGP-CK2.1 (+9.4%/day) release value of the following concentrations were (50 nM) 5 nM/day, (100 nM) 10 nM/day, (300 nM) 30 nM/day, and (500 nM) 50 nM/day (Fig. 1c). Single treatment of HGP-CK2.1 for 7 days at the given concentrations on C3H10T1/2 micromass cultures demonstrated the best chondrogenic activity at 5 nM/day concentration as shown using Alcian blue staining.

### Intra-articular injections of HGP-CK2.1 restored cartilage homeostasis in DMM mice

**Fig. 2:**
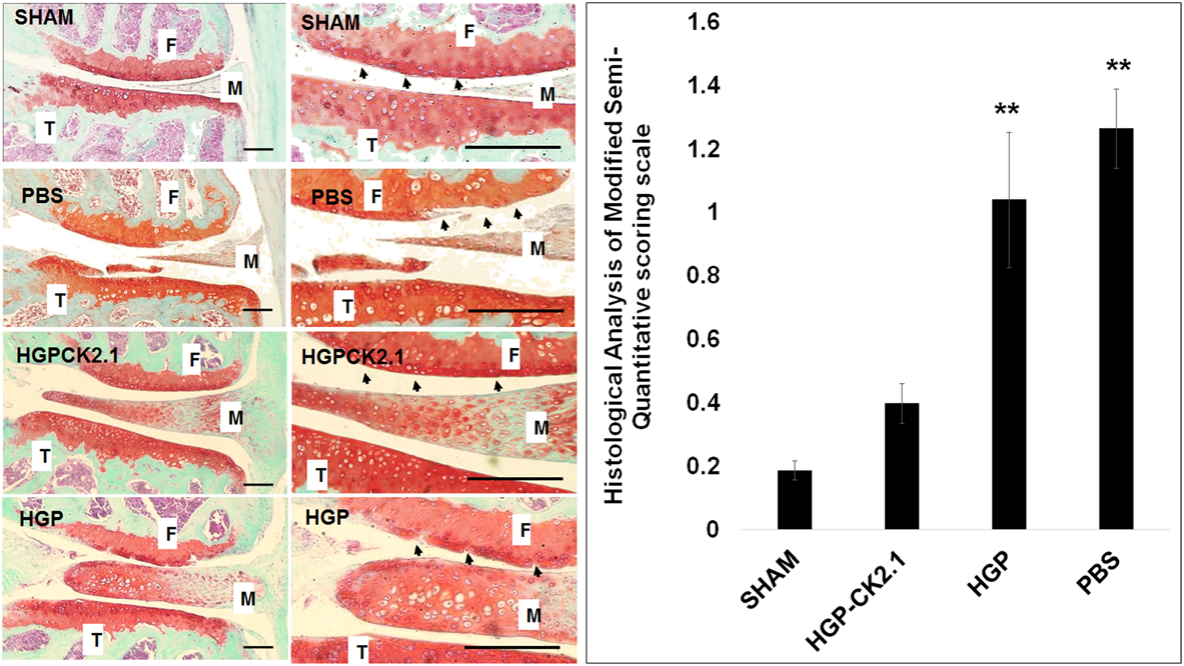
Intra-articular injections of HGP-CK2.1 induces articular cartilage repair in DMM mice. Mice were injected intra-articularly with PBS or HGP-CK2.1 (6 μM) or HGP 6 weeks post-DMM surgery (*n* = 6/group) and were compared to sham-operated mice. Sample sections were stained using Safranin O (*red*) and fast green (*turquoise*), and scoring was performed in four compartments of the knee using a modified semiquantitative scoring scale [19]. Mice injected with HGP-CK2.1 showed the greatest cartilage repair compared to the sham-operated group. *Arrows* indicate the area of cartilage damage. *Scale bars* = 50 μm. Error bars represent SEM; **p < 0.01. *F* femur, *HGP* hydrogel particle, *M* meniscus, *PBS* phosphate-buffered saline, *SHAM* sham-operated, *T* tibia

Surgical destabilization of the medial meniscus model is a technique that allows AC lesion formation at the weight-bearing regions of the medial tibial plateau and medial condyles [14]. This best reflects OA-like development in cartilage damage making this the ideal model for our study in mice. Destabilization of the medial meniscus was performed on male C57BL/6 J mice (*n* = 6/group) at age 10 weeks to induce OA-like changes. At 6 weeks post-surgery, the mice were injected with PBS, 6 μM of HGP-CK2.1 (adjusted based on the total amount of CK2.1 peptide released relative to the initial loading), or HGP alone intra-articularly once every 2 weeks. The concentration of HGP-CK2.1 at 6 μM was chosen based on peptide loading data that show release of CK2.1 on a 500 nM per day basis. These mice femur samples were processed and scored for cartilage damage using Mankins modified semiquantitative analysis [20]. Histological scoring and analysis demonstrated that mice injected with HGP-CK2.1 had the least OA damage and greater cartilage repair compared to those injected with HGP alone or PBS, compared to the sham-operated group (Fig. 2). Of note, mice injected with PBS alone had the greatest score for damage as seen by deeper fissures and loss of proteoglycan content.

### HGP-CK2.1-injected DMM mice had increased expression of collagen type II and type IX

**Fig. 3:**
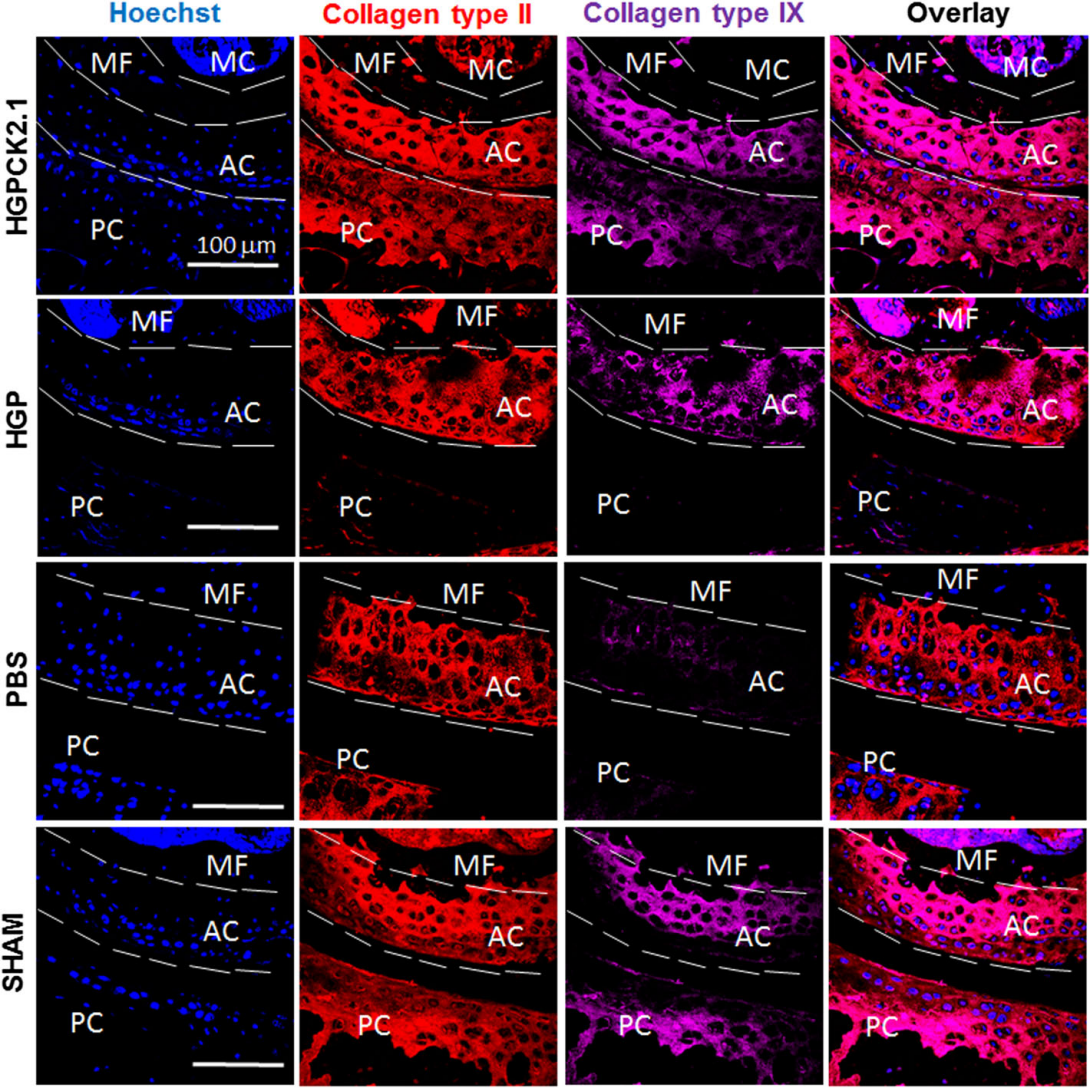
HGP-CK2.1 induced collagen type II and collagen type IX expression in AC. DMM mice injected with PBS or HGP-CK2.1 or HGP and sham-operated mice were immunostained for collagen type II (*red*) and type IX (*magenta*), and Hoechst (*blue*) was used to counterstain the nucleus of the residing cell and location. HGP-CK2.1 injected mice demonstrated high levels of collagen type IX compared to both HGP or PBS injected control mice. *Scale bars* = 100 μm. *AC* articular cartilage, *HGP* hydrogel particle, *MF* medial femur, *MC* marrow cavity, *PBS* phosphate-buffered saline, *PC* patellar cavity, *SHAM* sham-operated

Histological analysis demonstrated that the HGP-CK2.1-injected DMM mice exhibited the best restoration of cartilage in comparison to HGP- or PBS-injected mice. To identify the ECM composition of the newly regenerated cartilage, we immunostained the cartilage of DMM knee samples for collagen type II and type IX. Analysis of DMM knees injected with HGP-CK2.1 demonstrated collagen type II production that was consistent when compared to HGP- or PBS-injected mice. However, HGP-CK2.1-injected samples demonstrated elevated levels of collagen type IX in comparison to HGP- or PBS-injected mice (Fig. 3). This finding of elevated levels of collagen type IX in AC was consistent with our previously reported in vivo work [13].

### Increased expression of collagen type X and osteocalcin expression in HGP- and PBS-injected mice but not in HGP-CK2.1-injected mice

**Fig. 4:**
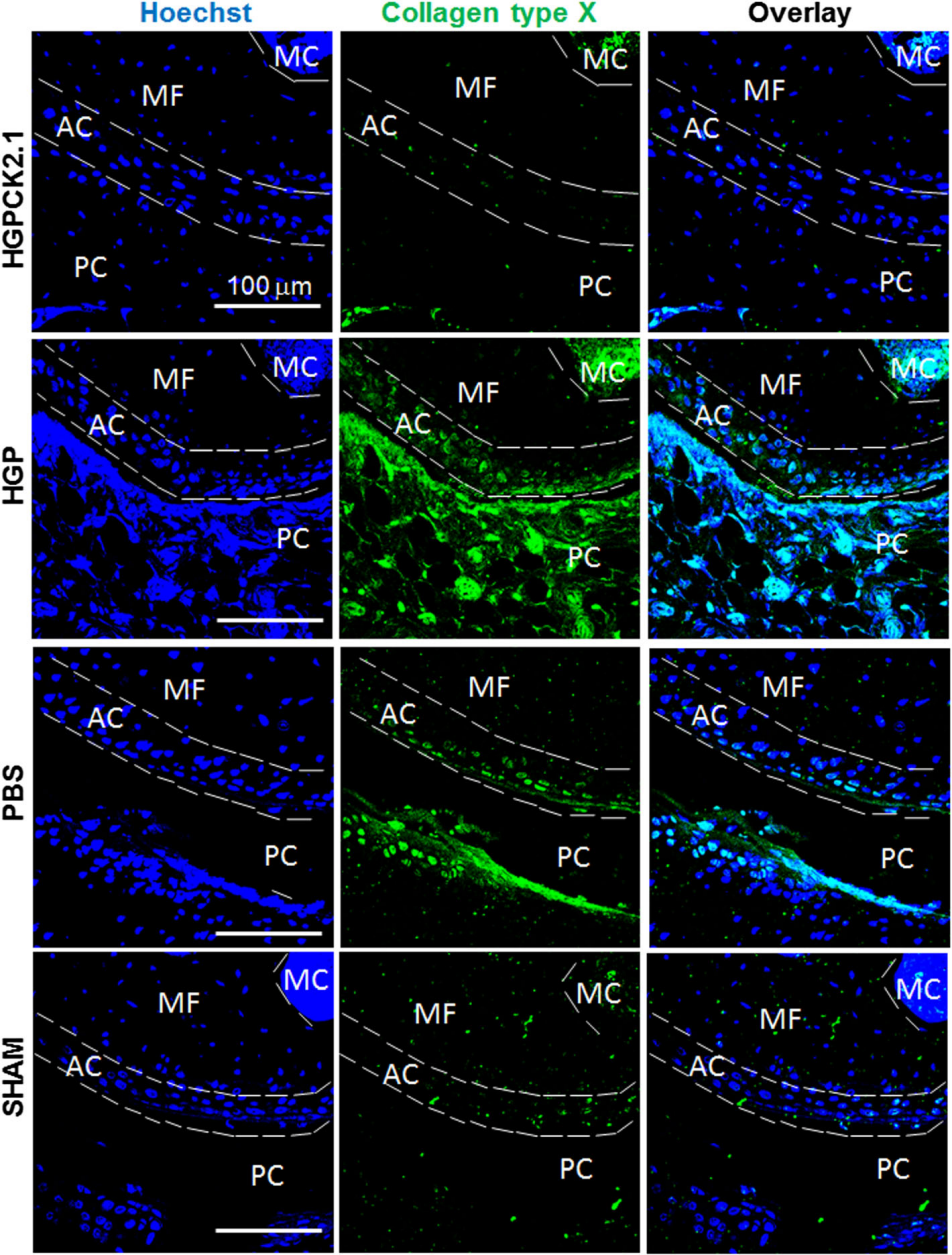
DMM mice injected with PBS and HGP induced collagen type X expression in articular cartilage but HGP-CK2.1 did not. DMM mice injected with PBS or HGP-CK2.1 (6 μM) or HGP and sham-operated mice were immunostained for collagen type X (*green*), and Hoechst (*blue*) was used to determine the nucleus of the residing cell and location. Immunostaining demonstrates increased collagen type X expression in the AC of PBS- and HGP-injected mice but not HGP-CK2.1-injected mice. *Scale bars* = 100 μm. *AC* articular cartilage, *HGP* hydrogel particle, *MF* medial femur, *MC* marrow cavity, *PBS* phosphate-buffered saline, *PC* patellar cavity, *SHAM* sham-operated

**Fig. 5:**
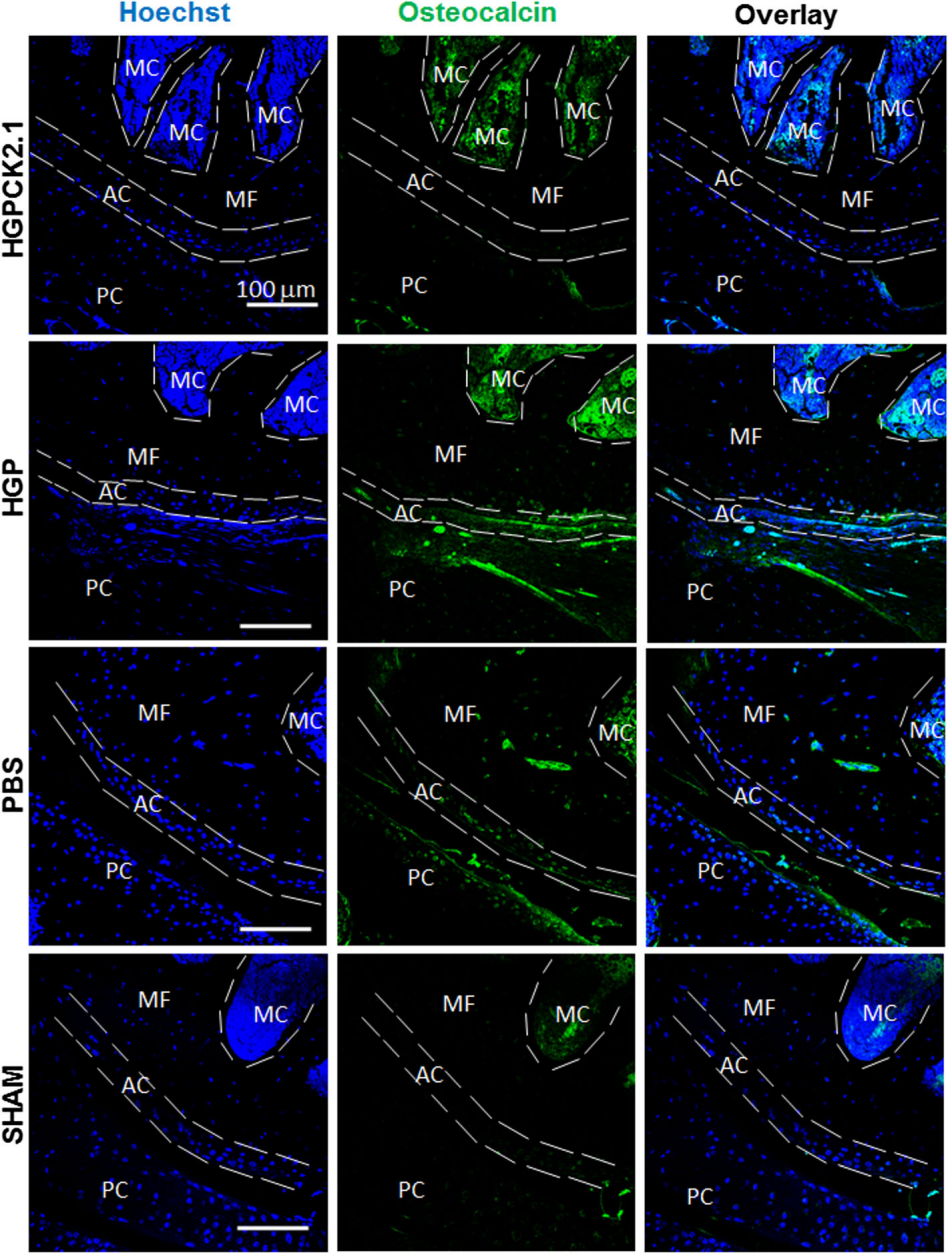
DMM mice injected with PBS showed increased osteocalcin expression but not those injected with HGP-CK2.1. DMM mice injected with PBS or HGP-CK2.1 (6 μM) or HGP and sham-operated mice were immunostained for osteocalcin (*green*), and Hoechst (*blue*) was used to determine the nucleus of the residing cell and location. Immunostaining demonstrates increased osteocalcin expression in AC of PBS-injected mice but not HGP-CK2.1-injected mice. *Scale bars* = 100 μm. *AC* articular cartilage, *HGP* hydrogel particle, *MF* medial femur, *MC* marrow cavity, *PBS* phosphate-buffered saline, *PC* patellar cavity, *SHAM* sham-operated

HGP-injected mice exhibited a moderate cartilage regeneration compared to PBS, while a significant increase in collagen type X in HGP-injected samples was observed similar to that of PBS-injected mice. However, HGP-CK2.1-injected samples showed a low expression of collagen type X in the regions of cartilage matrix repair (Fig. 4). This finding was previously reported in our in vivo work [13]. In addition, HGP-CK2.1 DMM mice samples did not demonstrate osteocalcin expression, as was observed in PBS injected mice samples (Fig. 5).

## Discussion

Our previous work demonstrated the potency of CK2.1-induced chondrogenesis in vitro and cartilage formation in vivo [13]. Here, we demonstrated the ability of this novel peptide for cartilage repair and cartilage tissue regeneration. In this study, a hydrogel particle-based delivery system was developed for the sustained release of CK2.1 peptide (HGP-CK2.1) to effectively stimulate cartilage regeneration. Here, we subject mice to DMM surgery (OA model) and these mice were allowed to sustain injury 6 weeks post-surgery to create OA-like conditions [14]. Treatment with HGP-CK2.1 over a 4-week period demonstrated the greatest cartilage repair compared to sham-operated mice. Analysis of collagen type II showed a correlation between cartilage repair and ECM formation. However, the greatest difference seen with HGP-CK2.1 treatment and the HGP- and PBS-injected groups was demonstrated in the differences between collagen type IX and type X. HGP-CK2.1-injected mice femurs demonstrated a greater collagen type IX expression but not collagen type X, whereas HGP- and PBS-injected mice exhibited high levels of collagen type X but not type IX. These data are consistent with the previously reported data on mice systemically injected with CK2.1 that induced collagen type IX expression in the AC of systemically injected mice [13]. Increased collagen type IX production, along with collagen type II, in mice injected with HGP-CK2.1 shows the clear restoration of AC tissue ECM components.

OA is characterized by a slow progressive degeneration of the cartilaginous tissue. This involves the disruption of structural and mechanical integrity organized around proteoglycans and the collagen framework of collagen type II, type IX, and type XI fibrillar structures [7]. Surgical destabilization of the medial meniscus in animals serves as the OA model [14]. Therefore, we used DMM surgery to induce OA-like damage to the AC. However, while instability models like the DMM demonstrate significant damage to the AC, it must be noted that the internal cellular OA-like mechanisms may be not be replicating the natural occurrence of the disease during joint aging. These OA animal models tend to develop susceptibility towards abnormal biomechanical loading following joint trauma including regenerative changes such as subchondral bone remodeling or osteophyte formation [14]. However, the DMM model provided the best reproducibility and progressive degradation of AC emulating the OA conditions [14]. For this reason, the DMM model was used in this study. Moreover, to minimize the number of intra-articular injections of the CK2.1 peptide, we employed a controlled release system at the localized site of injury.

HA is among the most commonly found proteoglycan macromolecule that is considered for clinical usage as viscosupplement for joint mobility enhancement [23, 24]. However, HA alone cannot induce cartilage repair, although it does provide temporary alleviation of pain at the knee [25]. Recently, HA-based hydrogels containing covalently integrated soft and deformable drug depots were developed to release therapeutic molecules in response to mechanical forces [26]. Intra-articular injection of HGP-CK2.1 provided us with a minimally invasive procedure for the delivery of the therapeutic peptide. Such a particle-based formulation enables a sustained release for the enhancement of tissue regeneration. In vitro testing of the HGP-CK2.1 demonstrated positive chondrogenic potency. Previously reported in vitro data on C3H10T1/2 micromasses stimulated with CK2.1 resulted in chondrogenic differentiation according to dose increase [13]. However, HGP-CK2.1-stimulated micromasses exhibit higher chondrogenic response at lower concentrations. This response could be a due to continuous release of the peptides affecting peptide localization rate and receptor availability, some of the mechanistic variables that should be investigated in our future studies. Although in vitro results demonstrate differences in chondrogenic activity among various concentrations, it is clear that in vivo results indicate a significant cartilage restoration in these DMM mice injected with HGP-CK2.1. The regenerated cartilage tissue demonstrated a higher content of collagen type II and type IX in HGP-CK2.1-injected mice compared to HGP- or PBS-injected mice. Collagen type IX and XI are necessary to form covalent cross fibrillation to provide the tensile strength [27, 28]. These data are consistent with our previous study, where an increased expression of collagen type IX in AC was observed in mice injected with the peptide CK2.1 systemically via the tail vein [13]. Furthermore, in OA, chondrocyte hypertrophy is a hallmark event. This terminal differentiation of chondrocytes is marked by the production of collagen type X. A transition from the production of collagen type II and IX to collagen type X necessitates chondrocyte hypertrophy and leads to cartilage degradation. In our study, we observed that HGP- and PBS-injected mice demonstrated higher levels of collagen type X expression, but not in HGP-CK2.1-injected mice. This was in accordance with our previously reported in vivo data from CK2.1 systemically injected mice [13]. In addition, expression of osteocalcin and osteophyte formation following cartilage degradation is commonly observed in OA-like conditions. This phenomenon is shown to be aggravated with excess mechanical forces and the influence of surrounding growth factor signaling such as that of BMP2 on OA [29, 30]. Our evaluation of mice injected with HGP-CK2.1 did not demonstrate an increase in collagen type X or osteocalcin levels. Furthermore, HGP-CK2.1-injected mice did not demonstrate any visible adverse side effects. This was similar to our previously reported in vitro data that showed no loss of cell number due to cellular death, or our in vivo systemic administration of CK2.1 that resulted in no adverse effects in mice [13]. However, there are many questions yet to be answered in our future work; for instance, the metabolic activity and mechanism by which the peptide actuates this particular process, or the dose-dependent effects of CK2.1 activity on AC. Similarly, we need to understand the signaling factors activated by the CK2.1 peptide downstream of BMPRIa that differ from BMP signaling that could contribute to this reparative process. Taken together, our data suggest that this novel peptide, CK2.1, initiates regeneration of damaged cartilage without the induction of chondrocyte hypertrophy, and may be a promising therapeutic candidate for the treatment of OA-like conditions.

## Conclusions

OA is an idiopathic cartilage degenerative disease. Currently, there are no drugs that can repair the lost cartilage. Utilizing the growth factor signaling pathways, researchers are attempting to address this issue. However, growth factors such as BMP are pleiotropic in nature and are known to enhance chondrocyte differentiation and chondrocyte hypertrophy. We designed a novel peptide, CK2.1, that activates the BMPRIa in the absence of the ligand [31, 32]. Our previous study demonstrated the potency of CK2.1-induced chondrogenesis in vitro and cartilage growth in vivo [13]. We demonstrate in this study the potency of CK2.1 for cartilage repair in an OA mouse model that was comparable to sham-operated mice without the induction of chondrocyte hypertrophy. These results are in accordance with our previous study [13]. Therefore, we have a unique opportunity to understand the signaling pathways that contribute to cartilage formation and cartilage repair. This peptide, CK2.1, may also be exploited for future therapeutic development in treating cartilage degenerative diseases.

## Abbreviations

AC: Articular cartilage
BMP: Bone morphogenetic protein
BMPRIa: Bone morphogenetic protein receptor type Ia
CK2: Casein kinase 2
DMEM: Dulbecco’s modified Eagle’s medium
DMM: Destabilization of the medial meniscus
ECM: Extracellular matrix
FBS: Fetal bovine serum
HA: Hyaluronic acid
HGP: Hydrogel particle
LA: Lactic acid
NHS: N-hydroxysuccinamide
OA: Osteoarthritis
PBS: Phosphate-buffered saline

## References

[1] Poole CA (1997). Articular cartilage chondrons: form, function and failure. J Anat.

[2] Allen KD, Golightly YM (2015). State of the evidence. Curr Opin Rheumatol.

[3] Sacks JJ, Luo YH, Helmick CG (2010). Prevalence of specific types of arthritis and other rheumatic conditions in the ambulatory health care system in the United States, 2001–2005. Arthritis Care Res (Hoboken).

[4] Murphy L, Helmick CG (2012). The impact of osteoarthritis in the United States: a population-health perspective: a population-based review of the fourth most common cause of hospitalization in U.S. adults. Orthop Nurs.

[5] Buckwalter JA, Mankin HJ, Grodzinsky AJ (2005). Articular cartilage and osteoarthritis. Instr Course Lect.

[6] Eyre DR, Weis MA, Wu JJ (2006). Articular cartilage collagen: an irreplaceable framework?. Eur Cell Mater.

[7] Poole AR, Rizkalla G, Ionescu M, Reiner A, Brooks E, Rorabeck C, Bourne R, Bogoch E (1993). Osteoarthritis in the human knee: a dynamic process of cartilage matrix degradation, synthesis and reorganization. Agents Actions Suppl.

[8] Blaney Davidson E, Vitters E, van Lent P, van de Loo F, van den Berg W, van der Kraan P (2007). Elevated extracellular matrix production and degradation upon bone morphogenetic protein-2 (BMP-2) stimulation point toward a role for BMP-2 in cartilage repair and remodeling. Arthritis Res Ther.

[9] Bragdon B, Bonor J, Shultz KL, Beamer WG, Rosen CJ, Nohe A (2012). Bone morphogenetic protein receptor type Ia localization causes increased BMP2 signaling in mice exhibiting increased peak bone mass phenotype. J Cell Physiol.

[10] van der Kraan PM, Blaney Davidson EN, van den Berg WB (2010). Bone morphogenetic proteins and articular cartilage: to serve and protect or a wolf in sheep clothing’s?. Osteoarthr Cartil.

[11] Bragdon B, Moseychuk O, Saldanha S, King D, Julian J, Nohe A (2011). Bone morphogenetic proteins: a critical review. Cell Signal.

[12] Bragdon B, D’Angelo A, Gurski L, Bonor J, Schultz KL, Beamer WG, Rosen CJ, Nohe A (2012). Altered plasma membrane dynamics of bone morphogenetic protein receptor type Ia in a low bone mass mouse model. Bone.

[13] Akkiraju H, Bonor J, Nohe A. CK2.1, a novel peptide, induces articular cartilage formation in vivo. J Orthop Res. 2016. doi:10.1002/jor.23342. 10.1002/jor.23342 PMC5522739 27312334

[14] Glasson SS, Blanchet TJ, Morris EA (2007). The surgical destabilization of the medial meniscus (DMM) model of osteoarthritis in the 129/SvEv mouse. Osteoarthr Cartil.

[15] Bragdon B, Thinakaran S, Moseychuk O, King D, Young K, Litchfield DW, Petersen NO, Nohe A (2010). Casein kinase 2 beta-subunit is a regulator of bone morphogenetic protein 2 signaling. Biophys J.

[16] Ge C, Xiao G, Jiang D, Franceschi RT (2007). Critical role of the extracellular signal-regulated kinase-MAPK pathway in osteoblast differentiation and skeletal development. J Cell Biol.

[17] Sahoo S, Chung C, Khetan S, Burdick JA (2008). Hydrolytically degradable hyaluronic acid hydrogels with controlled temporal structures. Biomacromolecules.

[18] Bruce SJ, Butterfield NC, Metzis V, Town L, McGlinn E, Wicking C (2010). Inactivation of Patched1 in the mouse limb has novel inhibitory effects on the chondrogenic program. J Biol Chem.

[19] Kang QKLJC, Gruber HE, An YH (2003). Histological techniques for decalcified bone and cartilage. Handbook of histology methods for bone and cartilage.

[20] Srinivasan PP, McCoy SY, Jha AK, Yang W, Jia X, Farach-Carson MC, Kirn-Safran CB (2012). Injectable perlecan domain 1-hyaluronan microgels potentiate the cartilage repair effect of BMP2 in a murine model of early osteoarthritis. Biomed Mater.

[21] Nohe A, Keating E, Underhill TM, Knaus P, Petersen NO (2005). Dynamics and interaction of caveolin-1 isoforms with BMP-receptors. J Cell Sci.

[22] Nohe A, Petersen NO (2007). Image correlation spectroscopy. Sci STKE.

[23] Xu X, Jha AK, Harrington DA, Farach-Carson MC, Jia X (2012). Hyaluronic acid-based hydrogels: from a natural polysaccharide to complex networks. Soft Matter.

[24] Kirchner M, Marshall D (2006). A double-blind randomized controlled trial comparing alternate forms of high molecular weight hyaluronan for the treatment of osteoarthritis of the knee. Osteoarthr Cartil.

[25] Jazrawi LM, Rosen J (2011). Intra-articular hyaluronic acid: potential treatment of younger patients with knee injury and/or post-traumatic arthritis. Phys Sportsmed.

[26] Xiao L, Tong Z, Chen Y, Pochan DJ, Sabanayagam CR, Jia X (2013). Hyaluronic acid-based hydrogels containing covalently integrated drug depots: implication for controlling inflammation in mechanically stressed tissues. Biomacromolecules.

[27] Itoh K, Udagawa N, Katagiri T, Iemura S, Ueno N, Yasuda H, Higashio K, Quinn JM, Gillespie MT, Martin TJ (2001). Bone morphogenetic protein 2 stimulates osteoclast differentiation and survival supported by receptor activator of nuclear factor-kappaB ligand. Endocrinology.

[28] Eyre D (2002). Collagen of articular cartilage. Arthritis Res.

[29] Christenson RH (1997). Biochemical markers of bone metabolism: an overview. Clin Biochem.

[30] van der Kraan PM, van den Berg WB (2012). Chondrocyte hypertrophy and osteoarthritis: role in initiation and progression of cartilage degeneration?. Osteoarthr Cartil.

[31] Bragdon B, Thinakaran S, Moseychuk O, Gurski L, Bonor J, Price C, Wang L, Beamer WG, Nohe A (2011). Casein kinase 2 regulates in vivo bone formation through its interaction with bone morphogenetic protein receptor type Ia. Bone.

[32] Moseychuk O, Akkiraju H, Dutta J, D’Angelo A, Bragdon B, Duncan RL, Nohe A (2013). Inhibition of CK2 binding to BMPRIa induces C2C12 differentiation into osteoblasts and adipocytes. J Cell Commun Signal.

